# The congressionally directed medical research programs’ Ovarian Cancer Academy: a new approach to training in biomedical research

**DOI:** 10.1017/cts.2022.404

**Published:** 2022-05-19

**Authors:** Tasha R. Wyatt, Lara Stepleman, Taylor Coleman, Leslie Robinson, Karen Wylie, Douglas A. Levine, Nita J. Maihle

**Affiliations:** 1 Uniformed Services University of the Health Sciences, Bethesda, MD, USA; 2 Medical College of Georgia at Augusta University, Augusta, GA, USA; 3 Cancer Center and Research Institute, University of Mississippi Medical Center, Jackson, MS, USA; 4 Department of Defense, Congressionally Directed Medical Research Programs, Frederick, MD, USA; 5 Perlmutter Comprehensive Cancer Center, NYU Langone Health, New York, NY, USA

**Keywords:** Mentoring, evaluation, ovarian cancer, early career investigators, communities of practice

## Abstract

**Introduction:**

Researchers have begun to change their approach to training in the biomedical sciences through the development of communities of practice (CoPs). CoPs share knowledge across clinical and laboratory contexts to promote the progress of clinical and translational science. The Congressionally Directed Medical Research Programs’ (CDMRP) Ovarian Cancer Academy (OCA) was designed as a virtual CoP to promote interactions among early career investigators (ECIs) and their mentors with the goal of eliminating ovarian cancer.

**Methods:**

A mixed-methods approach (surveys and interviews) was used to evaluate the effectiveness of the OCA for the eight ECIs and five mentors. Quantitative analysis included internal reliability of scales and descriptive statistics for each measure, as well as paired sample *t*-tests for Time 1 and Time 2. Qualitative data were analyzed for themes to discern which aspects of the program were useful and where more attention is needed.

**Results:**

Preliminary analyses reveal several trends, including the importance of training in grant writing to the ECI’s productivity, as well as the value of peer mentorship.

**Conclusion:**

The results show that the OCA was an innovative and effective way to create a CoP with broad implications for the field of ovarian cancer research, as well as for the future of biomedical research training.

## Introduction

As the need for investigators in translational and team-based science increases, so has an interest in developing mentorship programs to support early career investigators (ECIs), who are often defined as being within the first 3 years of their first faculty position (or equivalent) [1,2]. Mentoring is the cornerstone of such programs because it provides targeted support to ECIs as they work to become independent investigators [2,3]. As such, the majority of published reports about faculty training in clinical and translational science focus on practical aspects of mentorship, such as mentor–mentee relationships [4], developing mentorship skills [5], and mentee outcomes [6].

However, the world of clinical and translational science is slowly changing as researchers have started to reimagine collaborating within virtual communities of practice (CoPs). Traditional CoPs were designed as groups of people who met face-to-face [7], but the availability of online tools has facilitated the emergence of new forms of information sharing that have allowed CoPs to evolve into virtual communities [8]. Such CoPs promote flexible collaboration across geographic distances and distinct institutions among individuals who share a common interest [9]. They provide an environment that facilitates opportunities to share experiences, develop and discuss areas of interest, and build a sense of community. As researchers have learned to work within the COVID pandemic, virtual CoPs have become a more familiar way of supporting such efforts.

The purpose of this paper is to describe the preliminary qualitative and quantitative evaluation data collected during the period 2015–2020 for a new mentorship program devoted to the development of a community of ovarian cancer investigators by CDMRP’s Ovarian Cancer Research Program (OCRP), known as the Ovarian Cancer Academy (OCA). This academy was intentionally designed as a virtual CoP to support ECIs during the nascent phase of their independent research careers, while focusing on basic, translational, and clinical aspects of ovarian cancer research. Evaluation of this program began in 2015 with the goal of collecting data to facilitate improvements in the OCA and also to promote the advancement of ovarian cancer research. By providing a description of how the OCA’s virtual mentoring program was designed, as well as by sharing preliminary evaluation data collected on an early cohort in the program, it is hoped that other biomedical research training programs will be inspired to design their own virtual CoP as an alternative to more traditional mentorship programs.

### The Ovarian Cancer Academy (OCA)

In 2009, the Ovarian Cancer Research Program of the Congressionally Directed Medical Research Program within the U.S. Army Medical Research and Development Command of the Department of Defense (OCRP, CMDRP, USAMRC, DOD) initiated a new biomedical research training program, the Ovarian Cancer Academy (OCA). The OCA was created in recognition of the need to increase the number of investigators committed to ovarian cancer research. ECIs selected for participation in this Academy were selected for their past research contributions to the field of ovarian cancer research, as well as based on their proposed plans for future innovative research in the field of ovarian cancer.

The OCA is unlike other programs, many of which focus solely on supporting mentees through the development of mentor/mentee relationships [3]. Rather, the OCA was designed as a CoP to promote interactions among ECIs and their mentors, as well as with other established ovarian cancer researchers and ovarian cancer patient advocates, who were women who had past and/or current ovarian cancer. Each ECI had the opportunity to choose up to two mentors; one from their own institution and another from a different institution. The role of the mentors was to provide guidance to the mentee, both in the research lab and in the mentee’s career progression.

The ECIs choose their mentor(s) based on their professional needs. The ECIs wrote their mentors into their grants as co- or sub-PIs to provide protected time for mentorship and career guidance. The OCA did not formally evaluate how ECIs made decisions about who to invite as mentors; however, several possible reasons were given to ECIs in making this decision including ensuring professional compatibility, mentor’s expertise in the area of ovarian cancer research, mentor’s ability to help the ECI navigate their home institutions, and name recognition in the field of ovarian cancer research. In each case, it was the responsibility of the ECI to choose their mentors and determine when and how they would meet to achieve the goal of securing external funding.

The OCA’s basic design included monthly meetings where mentors, mentees, invited guests and speakers meet via Webex, alongside annual meetings, where ECIs were trained in grantsmanship and other professional skills. Being virtual has allowed the program to leverage interactions with internationally recognized scientists, a design recommendation shared by other clinical and translational science mentoring programs [10]. During typical monthly meetings, two ECIs presented their independent research and then discussed ways to interpret and extend their data. The ECIs also discussed how their work may relate to studies by others in the field, including those of other OCA participants. To promote such interactions, time was set aside to discuss potential ‘team science’ projects and various professional development training topics.

## Methods

This preliminary evaluation was designed as a mixed-methods study, which is considered an appropriate design for virtual CoPs [9] and was reviewed and approved by our university’s Institutional Review Board prior to data collection.

### Participants

Thirteen individuals, representing the first OCA cohort (*n* = 8) and their primary mentors (*n* = 5), were included in this study. The cohort mentees were 52% female, 62% Asian, and 75% assistant professors with a mean age of 43 years (*SD* = 3.92). Of the five mentors in the sample, 60% were male, 80% were White, and 80% were full professors at the onset of the study. Additional demographics are presented in Table 1.

**Table 1.** Mentor–mentee demographics

Demographic	*n (%) mentees*		*n (%) mentors*		Mean	Std Dev
Gender						
Male	2	(25%)	3	(60%)		
Female	5	(62.5%)	2	(40%)		
Missing	1	(12.5%)	–	–		
Race						
White	3	(37.5%)	4	(80%)		
Asian	5	(62.5%)	2	(20%)		
Missing	–	–	–	–		
Rank						
Assistant	6	(75%)	–	–		
Associate	1	(12.5%)	–	–		
Full^*^	–	–	5	(100%)		
Missing	1	(12.5%)	–	–		
# of years for mentees since last degree earned					8.14	1.57

* One mentor identified as an associate professor at the onset of the study and was later promoted.

### Procedure

For quantitative data collection, mentors and mentees were consented via Qualtrics, a cloud-based survey-building program. Written consent was waived for this study given the virtual format of the program. In October 2015, coincident with the start of a new leadership team and programming (Maihle and Levine), mentees were given their first survey (baseline) and were subsequently administered a survey every six months thereafter for 5 years, totaling 11 surveys during their OCA participation. Anonymous links to the survey were distributed to participants via email. Mentee surveys contained 12 metrics to evaluate the effectiveness of the program. Surveys took approximately 45 min to complete. Mentor evaluations began in October of 2016, one year into the OCA program for a total of 9 survey time points. Mentors were given an abbreviated survey version that assessed only their mentoring role and program satisfaction. In addition to these surveys, we also collected data on publications and grant funding obtained by this mentee cohort over the last 5 years.

For qualitative data collection, exit interviews were completed with study participants in the summer of 2020, corresponding to the completion of the 5-year OCA program by the first mentee cohort. Exit surveys consisted of open-ended questions focusing on benefits and challenges, as well as expectations and results of the program. Each interview lasted approximately 30–45 min. Interviews were conducted virtually by an independent evaluator using Microsoft Teams. After each interview, the transcript was downloaded, cleaned for analysis, and then checked against the original video recording for accuracy.

### Instruments

In addition to demographics, the mentee survey contained 12 metrics to assess over time five major categories of variables germane to the goals of the OCA: psychosocial wellness, research skills, mentoring competency, and patient-centered research, as well as overall program satisfaction. Given the large number of variables, measures were chosen for their brevity, validity evidence when available, and relevance to the investigators’ objectives for participants in the OCA. On some occasions, items were deleted if they represented duplication between metrics. Information regarding instruments can be found in Table 2.

**Table 2.** Survey metrics

Survey Instrument Name *(Subscales)*	Construct Assessed	Number of Items	Possible Range (Possible Range per Item)	Example Item	Modifications Made	Validity Evidence
*Psychosocial Variables*						
Single Item Burnout Measure [13] (Adapted from the Physician Worklife Study) [12]	Physician burnout	1	1–5 (1–5)	Please choose the item that best describes your current experience of stress and burnout	N/A	Shown to measure similar constructs (R^2^ = 0.50) to the emotional exhaustion sub-scale of the Maslach Burnout Inventory [14], an established burnout measure used within the health care field [13].
GRIT	Grit, defined as “Perseverance and passion for long-term goals”	12	12–60 (1–5)	I have overcome setbacks to conquer an important challenge.	Reverse-ordered items 2, 3, 5, 7, 8 and 11.	The items from this measure exhibit good internal reliability (α = 0.85) and factor analyses of this measure supported the final 12 items chosen for the scale, which were divided into two factors (CFI = 0.83 and RMSEA = 0.11).
Job Satisfaction	Participants’ job satisfaction	1	1–5 (1–5)	Compared to what you think your job satisfaction should be, what is your overall level of job satisfaction?	N/A	Validity for this specific item was not detailed; however, single item global job satisfaction instruments have generally been found to be valid and as good as multi-item scales [14].
Psychological Well-Being	Physician well-being.	8	8–56 (1–7)	I lead a purposeful and meaningful life.	No changes were made to this scale.	The items for the Psychological Well-Being Scale have good internal reliability (α = 0.86) and the overall PWB scale correlated moderately to strongly with two other well-being scales (*r* = 0.69 and *r* = 0.80).
*Research Variables*						
Collaborative Leadership Scale (n.d.) *(Building Trust Subscale)*	Participants’ attitudes toward collaborating with other researchers.	10	10–50 (1–5)	I build communication processes that make it safe for people to say what is on their minds.	Original 7-point rating scale was changed to 5-point rating scale.	Not determined.
Cross-Disciplinary Collaboration Activities Scale	Frequency in which researchers participated in activities that were outside of their primary field of study.	6	6–35 (1–5)	I attend meetings or conferences outside of my primary field.	Statements were adapted to first-person point of view. Original 7-point rating scale was changed to 5-point rating scale.	This scale was found to have good internal reliability (α = 0.81) and to have a high correlation with the Research Orientation scale, in that participants in the original study were found to participate in less cross-disciplinary collaboration activities, while scoring higher on unidiscplinary items, and vice versa.
Research Orientation Scale *(Transdiciplinary items)*	Participant engagement in unidisciplinary, mulitidisciplinary, interdisciplinary transdisciplinary, research.	5	5–35 (1–7)	In my own work, I typically incorporate perspectives from disciplinary orientations that are different from my own.	Original 5-point rating scale was changed to 7-point rating scale.	The Research Orientation Scale exhibited an acceptable goodness-of-fit (CFI = 0.95, RMSEA = 0.00) and were found to have adequate internal reliability (α = 0.74).
*Mentoring Competency Variable*						
Mentoring Competency Assessment *(Maintaining effective communication, Aligning expectations, Assessing understanding, Addressing diversity, Fostering independence, Promoting professional development)*	Mentees’ perceived skill level of their research mentor and themselves as a research mentor.	26	26–182 (1–7)	Rate how skilled you feel you/your mentor is in the following areas: Active Listening.	No changes were made to this scale.	The items for the Mentoring Competency Assessment were found to have excellent internal reliability (α = 0.95) and acceptable goodness of fit (CFI = 0.87, RMSEA = 0.080). The MCA self-assessment also had excellent reliability (α = 0.91) and acceptable goodness of fit (CFI = 0.85, RMSEA = 0.069).
*Patient-Centered Research Variables*						
Attitudes about Patients, Families, and Consumers in Research (author-derived)	Participants’ attitudes toward involving patients and their families in the research process	4	4–28 (1–7)	I believe that patients and families bring a perspective to a research project that no one else can provide.	Statements were adapted to first-person point of view. One item was removed due to similarity to an item in another scale used.	Not determined.
Patients and Families Research Involvement Scale (author-derived)	Gauge consensus regarding the principles of successful consumer involvement in NHS (National Health Service) research.	9	9–63 (1–7)	I seek agreement between the research staff and patients and families involved in my research.	“Consumers” was changed to “patients and families.” Statements were adapted to first-person point of view. Item 6 was divided into 2 items.	The original study used a postal Delphi process, which was administered twice, to validate principles chosen to indicate successful consumer involvement in NHS research. Eight principles were ultimately validated, resulting in the current scale used.
Racial Disparities (author-derived)	The extent to which participants take into account racial disparities in their research.	1	1–7 (1–7)	In my research studies, I take into account racial disparities.	No changes were made to this item.	N/A
*Program Satisfaction*						
Program Satisfaction Survey (author-derived)	Participant satisfaction with OCA.	8	1–7 (1–7)	Program Director 1	No changes were made to this item.	N/A

### Analysis Plan

Quantitative survey analyses were completed and imported into IBM SPSS Statistics Software program (SPSS), version 26. Because of the large number of time points and small number of mentee participants (with missing participant data for some time points), we combined time points such that we compared “early program responses” (which was the actual first survey for all but one participant), labeled Time 1. Later time points used were the last, most complete set of responses we had from a participant, anywhere between 40 and 60 months which served as Time 2. Statistical analyses included internal reliability of scales and descriptive statistics for each of the measures, as well as paired sample *t*-tests for Time 1 and Time 2. Mentors’ quantitative data are not included for this initial cohort because of the (initial) low response from mentors; we anticipate including mentor data in future studies as more cohorts complete the program. Data on number of publications and number of grants and dollar amounts were extracted from NIH RePorter (https://reporter.nih.gov/) and PubMed (https://pubmed.ncbi.nlm.nih.gov/), respectively.

Qualitative interviews for mentors and mentees were imported into Dedoose, a cloud-based software program, to be analyzed by an independent evaluator using latent content analysis [11]. The data were analyzed for larger themes across both the mentors and mentees to discern which aspects of the program were useful and where more attention might be needed. The mentor responses were used to provide both context and depth in the interpretation of the mentee responses.

## Results

### Qualitative Analysis

The qualitative analysis indicates that the OCA program was highly valued by both mentors and mentees. Participants described the program as a way for mentees to become more intentional about their research, receive feedback from other experts working in the field, and to learn the skills they need to be successful within and beyond the laboratory or patient bedside. In the following sections, strengths of the OCA from the perspective of the ECIs are presented, as well as areas in which the program needs further development.

#### OCA strengths

Participants indicated that the OCA’s individual DOD CDMRP OCRP-sponsored funding (‘ECI Awards’) were helpful to their overall development because it allowed participants to focus on building a program of research without having to worry about funding. As one participant explained, “A lot of funding opportunities are much shorter, which makes it really hard to kind of settle in” (Participant3). Participants indicated that having a funded laboratory allowed them to refine their research goals, identify potential grants, and kick start their nascent research programs. Funding also helped expand their laboratory capacity, as this participant explained, “The funding has definitely been instrumental in shaping my lab, getting things done, and getting students” (Participant5). Participants also explained that not having to worry about securing additional funding for a period of time allowed them to focus on their own professional development as scientists. Mentors corroborated this finding indicating that many of the junior awards available to researchers are only for 2–3 years making the OCA funding incredibly helpful, “My mentee was able to write one small grant application and then be able to get [a] five year grant, which really made all the difference in terms of the successful career that he has now” (Participant9).

Additionally, being able to develop as a professional was an unanticipated benefit. Having reduced pressure to secure additional funding meant they could focus on the continued development of their research program and build a support network that would endure long after their participation in the program ended. For example, this participant explained, “I think that [what was most valuable] was both the funding aspect of it as an early career investigator and also the community aspect… I didn’t realize how strong the community would be at the time I was applying” (Participant3). Having a supportive community was important because it reduced isolation, but also provided technical support when participants needed it. One participant described it as “a family where we help each other” (Participant8), suggesting that both personal and professional relationships were created. Mentors also thought the community building was important because it created a safe and collaborative atmosphere for participants, which is a departure from other programs or scientific meetings where attendees compete with each other. In the OCA, there was no need for competition because everyone was there for the same purpose, to support the work of ECIs in ovarian cancer research, as one mentor explained, “The Academy is very ideal because everybody gets along so well. I mean, you see very little friction in the Academy meetings… [because] they’re all in the same mode there” (Participant12).

Participants also liked that while participating in the program their success was tracked, which helped them to reflect on what they have accomplished throughout the year and make plans for the following year. For example, one participant indicated she liked that the OCA tracked the grants she applied for, including ones that were funded and ones that were turned down. She also liked that the OCA was interested in her publications, committee work, and her other professional development activities. Again, this gave her a sense that there were others in her community tracking her development who could help her think through areas she may need to strengthen as an ECI moving toward independence.

Additionally, the ECIs problem-solved everyday issues in dealing with their institutions or departments, as this one shared, “[The OCA was valuable] to not just connect with [others] scientifically, but [to hear about] the nuts and bolts of how they were doing within their own institution’s department.” Another participant described how having a community was useful to quickly move through issues in ways that have the potential to impact productivity. Participating in a virtual CoP normalized communication in ways that were not available face to face, “We talk frequently [via] a chat room. Whoever has problems, technical issues, or you know, when we applied for grants, we face difficult questions, and so we talk to each other [about it]” (Participant8).

Finally, participants described the high-quality mentoring they received on how to conduct ovarian cancer research. As one participant explained, “People don’t really realize how much it means for us to have that kind of support and mentorship in place” (Participant6). Mentors took their roles seriously and spent considerable time supporting mentees in their research. The protected time they received from being a co- or sub-PI on the ECI’s grants helped ensure mentors could focus on the needs of the mentee, as this mentor explained, “It is nice to have a little bit of protected time, I take my mentoring very seriously and so I meet with all my mentees. I would say that 25% of my week right now is [spent mentoring]” (Participant13).

ECIs also expressed that they grew as mentors themselves. For example, one participant indicated that through the mentoring relationship they learned about the importance of being a team player. Whereas previously he did not take his role on a team as seriously as a leadership role, the OCA helped him realize the importance of this, “Even when you are not the leader of the group, you need to be a good team player [and] you need to care about the team” (Participant1). Others described learning about their responsibility to others in different ways. For example, another participant shared, “[The OCA] really taught me more about mentoring, how to become a better mentor, how to navigate interactions with mentees, and how to get the most out of interactions with mentors” (Participant6).

In summary, the OCA’s first cohort of ECIs to be evaluated found that the program’s funding, networking and community building, and also expert mentoring, were extremely helpful to their growth and development as ECIs in the field of ovarian cancer research. These results were substantiated by the participants’ mentors who felt that the program was successful in helping participants successfully launch themselves in the early phases of their research careers, as this one indicated, “The success rate is going to be pretty substantial, and I think it speaks to the role of the Academy in helping foster the early phases of career development for those individuals” (Participant11).

#### OCA areas of improvement

Although participants felt there were many areas of strength, a subtler theme showed the need for more skill development on grant writing. ECIs indicated that what was provided in the OCA was very helpful, as this one expressed, “the grant writing and grant review process is particularly strong” (Participant3). However, the mentors and mentees expressed concern that once they left the program, they wouldn’t be able to secure their own funding, even though several had already secured additional funding at the time of the exit interviews. They also expressed that conducting team science was challenging, given that they had their individual research projects they needed to attend.

Additionally, the inclusion of patient advocates in the OCA was perceived differently by physician vs. non-physician scientists. Some felt that these interactions were exceptionally rewarding because this was an aspect of ovarian cancer research for which they had little previous experience, such as this one, “We got a lot of opportunities to interact with consumers and patient advocates, which generally we don’t get. The OCA was a very good platform to get to know the ovarian cancer patients and what they went through” (Participant7). Mentors also felt that this was a benefit, explaining, “I’ve been in ovarian research for 15 years and actively involved in a number of meetings addressing ovarian cancer …to actually see the advocates, I think has been extremely valuable” (Participant12). However, other ECIs indicated they would like even more regular opportunities to interact with these community members because of how powerful their stories were in shaping their research, as this ECI shared, “I kind of want more interaction with the patients and patient advocates” (Participant6).

In summary, after participating in the OCA, participants thought that more opportunities for grant writing and grantsmanship development, with potentially even greater inclusion of patient advocates in activities of the program would be useful. Although these components were already included in the design of the OCA, participants felt they were beneficial to their career development and wanted even more opportunities to grow and learn from such experiences.

### Quantitative Analysis

#### Survey data

Internal reliability was calculated using Cronbach’s Alphas. Table 3 presents the reliability for each scale (using Time 1 data) and the means and standard deviations from for Time 1 and Time 2 data for each assessment. The minimum and maximum scores for each item and each scale, as well as the number of items per scale can be found in Table 2. Because the program satisfaction items assess different aspects of the program, item reliability is not calculated as these were used at the individual item level.

**Table 3.** Comparison of means for mentees at time 1 and time 2

Instrument	α	Mean Difference with 95% CI (LL, UL)	Time 1 Mean (Std dev)	Time 2 Mean (Std Dev)
Burnout	N/A	−0.16 (−0.96, 0.62)	4.33 *(SD* = *1.03)*	4.17 *(SD* = *0.75)*
Grit	0.53	−0.01 (−0.32, 0.33)	4.08 *(SD* = *0.28)*	4.07 *(SD* = *0.29)*
Job Satisfaction^*^	N/A	−0.50 (−0.38, 1.38)	3.83 *(SD* = *0.98)*	3.33 *(SD* = *0.82)*
Psychological Well-Being	0.97	0.04 (−1.20, 1.12)	6.13 *(SD* = *1.25)*	6.17 *(SD* = *0.42)*
Collaborative Leadership Scale-Building Trust Subscale	0.88	0.05 (−0.64, 0.54)	4.34 *(SD* = *0.58)*	4.39 *(SD* = *0.59)*
Cross-Disciplinary Collaboration Activities Scale	0.85	−0.23 (−0.47, 0.80)	3.70 *(SD* = *0.74)*	3.53 *(SD* = *0.39)*
Research Orientation Scale-Transdisciplinary Subscale^+^	0.89	0.16 (−0.89, 0.55)	5.77 *(SD* = *1.03)*	5.93 *(SD* = *0.74)*
MCA Mentee Assessment of Research Mentor	0.97	0.31 (−2.66, 2.04)	5.23 *(SD* = *1.18)*	5.54 *(SD* = *0.93)*
Maintaining effective communication	0.87	−0.20 (−2.08, 2.49)	5.80 *(SD* = *1.10)*	5.60 *(SD* = *1.34)*
Aligning expectations	0.93	0.25 (−3.60, 3.10)	4.95 *(SD* = *1.45)*	5.20 *(SD* = *1.98)*
Assessing understanding^*^	0.95	0.60 (−1.71, 0.51)	5.60 *(SD* = *0.89)*	6.20 *(SD* = *0.45)*
Fostering independence^*^	0.96	0.52 (−2.79, 1.75)	5.12 *(SD* = *1.63)*	5.64 *(SD* = *0.38)*
Addressing diversity	0.35	0.39 (−2.05, 1.27)	5.50 *(SD* = *1.11)*	5.89 *(SD* = *0.55)*
Promoting professional development^*^	0.91	0.56 (−3.61, 2.49)	4.76 *(SD* = *1.81)*	5.32 *(SD* = *0.38)*
MCA Mentor Self-Assessment	0.97	0.23 (−1.31, 0.85)	5.94 *(SD* = *0.94)*	6.17 *(SD* = *0.49)*
Maintaining effective communication	0.85	0.43 (−1.72, 0.86)	5.87 *(SD* = *1.00)*	6.30 *(SD* = *0.46)*
Aligning expectations	0.90	0.16 (−1.30, 0.98)	5.92 *(SD* = *0.97)*	6.08 *(SD* = *0.59)*
Assessing understanding	0.88	0.10 (−1.37, 1.17)	6.10 *(SD* = *0.89)*	6.20 *(SD* = *0.45)*
Fostering independence	0.88	0.08 (−0.88, 0.72)	6.12 *(SD* = *0.81)*	6.20 *(SD* = *0.47)*
Addressing diversity	0.85	0.30 (−1.73, 1.13)	5.70 *(SD* = *1.30)*	6.00 *(SD* = *0.61)*
Promoting professional development	0.86	0.24 (−1.42, 0.94)	5.88 *(SD* = *1.06)*	6.12 *(SD* = *0.50)*
Attitudes about Patients, Families, and Consumers in Research	0.87	0.14 (−1.03, 0.76)	5.83 *(SD* = *1.08)*	5.97 *(SD* = *0.69)*
Patients and Families Research Involvement Scale	0.93	0.45 (−1.63, 0.74)	5.00 *(SD* = *1.16)*	5.45 *(SD* = *0.58)*
Racial Disparities	N/A	0.00 (−0.94, 0.94)	5.17 *(SD* = *1.83)*	5.17 *(SD* = *1.47)*

*Note:* CI, confidence interval; LL, lower limit; UL, upper limit.

* These scales showed a notable change of .5 scale points or greater between Time 1 and Time 2.

+ These scales show a *P* < .10, indicative of a trend toward significance.

Because we used combined time points as detailed in the analysis plan, there was no missing data in Time 1 and Time 2 comparisons. Still, no statistical significance (*P* < 0.05) occurred between Time 1 and Time 2. This was anticipated given the small *n* for the first year. However, many measures displayed an increase in mean scores between Time 1 and Time 2 in the directions predicted. Table 3 summarizes the difference in scores between Time 1 and Time 2. In particular, we noted scales in which there was a .50 point or greater change. While not statistically significant, investigators, specifically the principal investigator and two evaluation team members, reached a consensus that this was a meaningful change worthy of further consideration. Table 4 presents the individual item data on program satisfaction.

**Table 4.** Evaluation by mentees of the Ovarian Cancer Academy (OCA) programs components

Item	Mean
Joint Research Project	4.17 *(SD* = *2.04)*
Early Career Investigator Presentations	6.33 *(SD* = *0.82)*
Incorporation of consumer advocates in the research process	5.33 *(SD* = *1.97)*
Program Director 1	6.83 *(SD* = *0.41)*
Program Director 2	6.83 *(SD* = *0.41)*
LinkedIn	2.80 *(SD* = *1.79)*
Ovariancanceracademy.org	4.20 *(SD* = *2.59)*
Webex	5.80 *(SD* = *1.10)*

*Note:* Items were scored on a 7-point scale, where 1 = Not At All Helpful and 7 = Extremely Helpful.

#### Publications and grant data

The total number of publications produced by the first OCA mentee cohort to be evaluated for the period 2015–2020 was 109, 60 of which an ECI served as the first or last author. The total number of NIH grants received was 7, accounting for 2.5 million total dollars.

## Discussion

This study analyzed evaluation data collected from a cohort of ECIs who participated in the recently established DOD CDMRP OCRP-sponsored OCA. The qualitative results indicate that protected funding, leadership-directed opportunities to network, and expert mentoring were very helpful to ECIs’ growth and development in the field of ovarian cancer research. Mentoring was particularly valuable for the ECIs because not only did the mentors help them grow as scientists, but they also modeled exemplary mentorship skills.

Both data sources also demonstrate that OCA participant responses to inclusion of patient advocates in OCA activities were dependent on the prior experience of the ECI. Physician-scientists engaged in on-going interactions with patients did not feel the need for more patient interactions, whereas non-physician scientists greatly appreciated this opportunity. As another potential area for improvement, ECIs reported the need for greater opportunities for grant writing and grantsmanship development, as well as more structure around team science expectations, a finding that was echoed in previous work showing that achieving team science is a challenging area in mentoring because various points of view need to be represented [12].

Taken together, these results suggest that the OCA’s virtual CoP is an effective means for providing ECIs with the peer support and mentoring they need to become successful as clinical and translational scientists. Earlier work on mentoring in clinical and translational science has shown that when a) institutional resources (e.g. infrastructure, financial resources), b) training (e.g. didactics, research experience), c) conflicting demands (e.g. clinical and service responsibilities), and d) relational factors (e.g. mentoring, networking) are addressed, ECIs experience both extrinsic success (e.g. promotions, funded grants) and intrinsic success (e.g. career satisfaction) [6]. Findings reported in this study show that with protected funding, training, and networking, ECIs were highly productive by traditional measures of success, and are also able to establish support to sustain their continued professional advancement.

In moving forward, the OCA will continue to collect data to evaluate the next cohort of ECI’s in this program, which is funded through 2025, with the potential for even longer-term support. The next CoP iteration will make design changes to the evaluation methods, including six-month interviews to explore how the careers of these ECIs change over time, including navigating obstacles and overcoming scientific and organizational challenges. Specifically, we will also explore how ECIs navigated obstacles as a result of the COVID-19 pandemic, asking how the pandemic affected their ability to adhere to a research plan, their outlook on future funding, and home and work-life balance, such as caring for children and/or ill loved ones.

Additionally, we would also like to make changes to the survey administration. Because the OCA was a new program, it was challenging to get participants to consistently respond to the surveys. The five mentors who were mentioned in this manuscript were the only five to complete every time point’s evaluations, whereas the others only responded intermittently. Therefore, in the next iteration, given the number of metrics included during the quantitative assessment, each metric will be reviewed to determine its overall contribution to understanding the ECIs’ experience in this program. The goal will be to shorten the survey’s length with the hope that more of them will be completed.

In another vein, although the OCA was intentional in recruiting patient advocates that would be helpful to the ECI’s research agenda, we did not fully evaluate the advocates’ experiences in the program. Rather, advocates were selected based on their commitment to ovarian cancer research and their willingness to help our scholars. In addition, they were chosen because many of them were interested in supporting racially underrepresented populations, or were racially underrepresented themselves. Initially, we viewed these advocates in a supporting role, but after the evaluation, we have reframed our view and realize that their experiences should be a part of the formal evaluation, both to gain a more holistic view of the program, as well as ensuring a sense of belonging. Stakeholder engagement is extremely important at every stage of translational science, and a bidirectional approach, in which knowledge is actively constructed, discovered, transformed, and extended between advocates and mentees equalizes the power hierarchy in translational work and makes for better science. Although the OCA award does not provide funding to train and orient patient advocates, going forward, we plan to give greater thought to how we might engage with advocates differently.

And finally, we noted that the same degree of racial underrepresentation that is seen in biomedical research [13], more generally is also seen in ovarian cancer research. One of the goals of the OCA is to overcome this disparity, which we hope to bring into focus in the next iteration of this academy. Along these lines, the OCA evaluation survey data showed that the number of ECIs who identify as racially underrepresented individuals has increased, and there is more research being conducted on ovarian cancer inequities. We feel that with the next iteration, we will be able to better support these individuals. And in making all of these changes, we hope that the OCA will be used to model effective evaluation for new mentoring programs interested in creating virtual CoPs for their target population.

## Disclaimer

This work was prepared by a military or civilian employee of the US Government as part of the individual’s official duties and therefore is in the public domain. The opinions and assertions expressed herein are those of the author(s) and do not necessarily reflect the official policy or position of the Uniformed Services University or the Department of Defense.
